# Pilot study of the productivity and *Salmonella* seroprevalence in pigs administered organic acids

**DOI:** 10.3389/fvets.2023.1123137

**Published:** 2023-03-03

**Authors:** Manuela Roldan-Henao, Anders Dalsgaard, Nora Cardona-Castro, Lina Restrepo-Rivera, Luis Carlos Veloza-Angulo, Lis Alban

**Affiliations:** ^1^Department of Veterinary and Animal Sciences, CES University, Medellin, Colombia; ^2^Department of Veterinary and Animal Sciences, University of Copenhagen, Copenhagen, Denmark; ^3^Instituto Colombiano de Medicina Tropical, CES University, Medellin, Colombia; ^4^Department for Food Safety and Veterinary Issues, Danish Agriculture & Food Council, Copenhagen, Denmark

**Keywords:** *Salmonella*, organic acids, pigs, seroprevalence, growth promoters, growth performance

## Abstract

Control of *Salmonella* in pig/pork production is important to protect public health because pork is one of the main sources of human infection. Moreover, antimicrobial use in pig farms should be kept low to minimize development and transmission of antimicrobial resistance. This pilot study evaluated the productivity and *Salmonella* seroprevalence in pigs administered organic acids (OA) compared to pigs given growth promoters in one farm in Antioquia, Colombia. Two groups each consisting of 60 pigs of 6-weeks of age were studied for 4 months. One group was provided feed and water with OA (Selko pH^®^ and Selacid^®^), whereas the other group (control) received antimicrobial growth promoters according to routine feeding practices (tylosin and zinc bacitracin). Blood samples were taken three times (T1–T3) and pigs were weighted five times to calculate daily weight gain (DWG) and feed conversion ratio (FCR). Initially when the pigs were 6 weeks old (T1), the *Salmonella* seroprevalence was 1.7% in both groups. When the pigs were 11 weeks old (T2), the seroprevalence was significantly lower in pigs provided OA compared to the control group (19 vs. 47%, *P* < 0.001), whereas when the pigs were 23 weeks old (T3), the seroprevalence did not differ between the groups (62 vs. 77%; *P* = 0.075). The cumulative DWG was significantly higher in the intervention group than in the control group (713 vs. 667 g/day; *P* < 0.001). The cumulative FCR did not differ between groups (2.80 vs. 2.77; *P* = 0.144). The pilot study indicates that cleaning the water pipes and administrating OA improve productivity in pigs and delay exposure to *Salmonella* spp. when compared with growth promoters. Thus, OA could replace antimicrobial growth promoters and reduce antimicrobial use and resistance. However, the study should be repeated before firmer conclusions can be drawn.

## 1. Introduction

Salmonellosis is a foodborne, zoonotic disease that is generally self-limiting (1). Worldwide, non-typhoidal *Salmonella* is ascribed to ~93.8 million human cases of acute gastroenteritis and 155,000 deaths annually (2, 3). In the United States, the cost of human salmonellosis is estimated to be around $2.9 billion per year (4). Denmark has carried out intensive programs to control *Salmonella* in the animal production chain since 1990s which has resulted in a low human incidence, i.e., 11.8 *Salmonella* cases per 100,000 habitants were registered in 2021 (5). In Colombia, human salmonellosis is underreported and considered an endemic disease with sporadic outbreaks. According to the Colombian National Institute of Health, 7,219 *Salmonella* cases were reported between 2000 and 2013 with *S*. Typhimurium (33.7%) being the most common serotype detected followed by *S*. Enteritidis (28.6%), *S*. Dublin (3.3%) and *S*. Derby (2.1%) (6, 7).

The distribution of the different *Salmonella* serotypes varies according to food source and geographical area (6). Infection with *S*. Enteritidis is often associated with consumption of eggs and poultry meat, whereas the other globally important serotype *S*. Typhimurium is related mainly to consumption of pork (6, 8). In 2020, 13.0% of the human cases of salmonellosis reported in the European Union (EU) were due to consumption of contaminated pork. In 2015, the *Salmonella* prevalence was 28.2% on pork carcasses at abattoirs in Colombia with *S*. Typhimurium, *S*. Agama and *S*. Agona being the main serotypes found (9). Comprehensive control of *Salmonella* throughout the food value chain can decrease the incidence of human salmonellosis (6, 10, 11).

Subclinical salmonellosis in pigs constitutes a source of *Salmonella*. After weaning, the pigs excrete *Salmonella* and infect other pigs in the pen. Excretion of *Salmonella* may increase at times of stress such as during transport to the abattoir and in the lairage area, resulting in high risks for contamination of the carcasses during slaughter unless adequate measures are taken (11–14). In Colombia, the between-farm *Salmonella* seroprevalence was 42.9% (*n* = 350) in 2020 in the seven main pig producing provinces (8, 15–17). Half of the *Salmonella* strains tested (*n* = 41) showed concurring resistance to penicillin, cefuroxime, tetracycline and trimethoprim/sulfamethoxazole. These types of antimicrobial resistances (AMR) in *Salmonella* may be ascribed to the routine use of antimicrobials supplemented to pig feed used in Colombia (3, 18) and is of concern for effective treatment of human salmonellosis (16). Moreover, reduced AMR levels are not just of benefit to human health but will also ensure that pigs suffering from diseases caused by bacterial pathogens can be treated.

Although the use of antimicrobial growth promoters is banned in some parts of the world including the EU, they are still allowed and commonly used in Colombia for disease control and to improve growth in livestock. However, use of such growth promoters leads to development of AMR. For this reason, alternatives have been sought to replace the antimicrobial growth promoters including preventive measures focusing on improving the health of pigs while maintaining productivity (19). Without maintenance of productivity, the farmers cannot be expected to change habits and replace antimicrobial growth promoters with alternatives.

Organic acids (OA) can be used to control *Salmonella* and promote growth in pigs. When administrated in water and/or feed, the OA cause a decreased pH to 3.8–4.2 at which the growth of many gastro-intestinal bacteria except lactobacilli is altered or directly inhibited. OA also modulate the intestinal fermentation patterns of feed creating a better gastro-intestinal environment with improved utilization of feed and growth (20–23). These positive effects of OA on feed conversion rate and growth performance are also described in poultry including increased egg production. The ability to decrease *Salmonella* colonization depends on the type of OA used (24). Although the antibacterial effect of OA is well known in theory, published results of efficacy in on-farm studies vary, with some reporting beneficial effects (25–28) while others fail to demonstrate any effect (29–31). Thus, further evidence is needed to establish at which concentrations and combinations OA could be used to control *Salmonella* spp. in pigs and to elucidate their effect on productivity (21, 32).

The purpose of this pilot study was to evaluate the effect of OA on the productivity and the *Salmonella* seroprevalence in pigs from weaning to slaughter. We undertook a clinical trial, comparing the effect of provision of OA with antimicrobial growth promoters in a pig farm in Antioquia, Colombia. The hypothesis was that OA supplements to water and feed were equally effective as the growth promoters. This would open up for a possible replacement of antimicrobial growth promoters with OA in line with the principles of prudent use of antimicrobials.

## 2. Materials and methods

### 2.1. Herd description

This study was endorsed by the Institutional Committee Cuidado y Uso de los Animales (CICUA) at the CES University, Medellin, Colombia (Code No. 206/Act No. 38). The study farm produced piglets as well as finisher pigs was selected in Antioquia, Colombia. The farm had a known positive status for *Salmonella*.

The farm had a total of 500 sows. The piglets were weaned at 4 weeks of age, where after they remained for 7 weeks in the weaning facilities. They were then moved to the growing facilities, where they would stay for 6 weeks. Finally, they were moved to the finishing pens where they remained for 6 weeks until slaughter. The feed was produced on the farm. The composition of the feed is shown in Supplementary Table S1. The pens were equipped with portable waterers to measure water consumption.

### 2.2. Baseline sampling

Prior to the start of the trial, sampling of blood, rectal swabs and fecal material was performed to determine the within-farm *Salmonella* seroprevalence and to confirm presence of *Salmonella* in the herd. In August 2020, blood samples and rectal swabs were taken from 10 lactating sows, 30 weaned piglets, 30 growing pigs and 30 finishing pigs. Subsequently in September and December 2020 as well as in April 2021, a total of 130 samples were collected, processed and analyzed in three different ways. The first 40 samples consisted of rectal swabs, which were transported in Selenite-Cystine medium (Instituto Colombiano de Medicina Tropical (ICMT), Medellin, Colombia) and processed at two different laboratories. The following 30 samples were fecal samples, each with a volume of around 25 g, and collected directly from the rectum of individual pigs to increase the sensitivity of the subsequent laboratory analysis. The fecal samples were placed in sterile plastic bags. The remaining 60 samples consisted of fecal swab samples which were transported in Aimes transport medium (ICMT, Medellin, Colombia).

### 2.3. Experimental design and sampling

**Figure 1:**
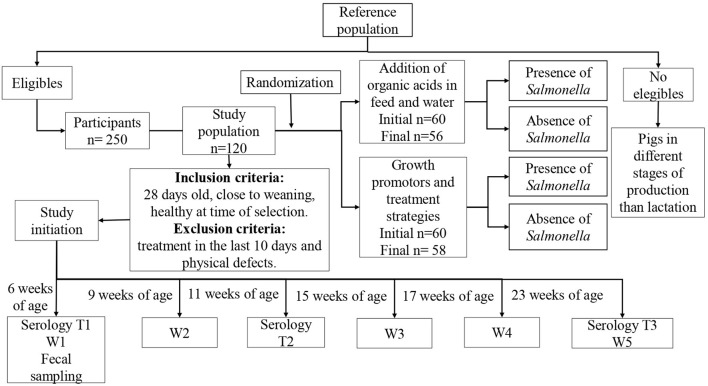
Study design. W1–5, weight 1 to weight 5.

A parallel, randomized, controlled clinical trial was performed at the selected pig farm including 120 individual pigs. The sample size was based on logistical and economic considerations. The piglets were randomly divided into two groups of 60 pigs each. Each individual pig was ear tagged with an identification number to ensure proper follow-up (Figure 1).

The inclusion criteria were piglets close to weaning at ~28 days of age and healthy at the time of sampling. The exclusion criteria were piglets that presented physical defects or that had received any antimicrobial treatment up to 10 days before the selection of the animals. At the time of sample selection, 250 piglets met the inclusion criteria and the formula K = *N*/*n* (K = sample interval, *N* = total population units and *n* = sample size) was used to determine the number of animals to be included in the study.

The farm veterinarian oversaw the assignment of pigs to each group in the pens before the sampling was initiated. Each pen was completely separated from other pens preventing the pigs in one group from having physical contact with other pigs. The trial started when the pigs were 6 weeks of age with a follow-up time of 4 months.

In September 2021, water samples were taken and microbiological and physicochemical analyses were performed to determine the dosage of Selko pH^®^ (Trouw Nutrition, Tres Cantos, Madrid) to be added to the water. These water quality analyses were done as the effect of Selko pH^®^ depends on the characteristics of the of water including pH, hardness, concentrations of minerals and organic matter as well as bacterial concentration. The results showed a high degree of fecal contamination of the water with an *E. coli* count of 1,944 CFU/100 ml, fecal coliforms of 3,888 CFU/100 ml, but with no isolation of *Salmonella* spp. It was therefore decided by the owner of the farm to disinfect the water pipes with 0.4 ml/l of citric acid solution (GREEN DAC^®^ ECOLAB, Bogota, Colombia) before beginning the clinical trial to ensure the effect of the OA treatment. Subsequent water samples obtained after cleaning the pipes contained 0 *E. coli* CFU/100 ml, 8 CFU/100 ml of fecal coliforms and absence of *Salmonella* spp. During the clinical trial, the pipes were cleaned every month using citric acid in the same way as described above.

The drinking water for the intervention group was supplemented with Selko pH^®^ that contains E 236 formic acid, E 260 acetic acid, E 295 ammonium formate, E 300 L-Ascorbic acid, E 330 citric acid, E 4 copper and E 6 zinc. Based on the water characteristics it was decided to add 0.8 ml/liter of Selko pH^®^ to the water to ensure the expected effect. This dosage was administered during the first 4 h of the day, every other day throughout the follow-up period. Likewise, Selacid^®^ (Trouw Nutrition^®^, Tres Cantos, Madrid) that contains E 200 sorbic acid, E 236 formic acid, E 260 acetic acid, E270 lactic acid, E 280 propionic acid, E 295 ammonium formate and E 330 citric acid was added to the feed. Two kg of Selacid^®^ per ton was added to weaner feed, whereas 1.5 kg per ton was used in grower and fattener feed during the entire study. The concentration of the individual compounds in the two commercial products were not declared and such information could not be obtained from the company. In the control group, tylosin phosphate 10% (1 kg per ton) was added to the weaner feed for the first 7 days of the study. Moreover, 15% zinc bacitracin (300 g per ton) was added to the grower feed for about 1 month.

Before starting the intervention with 6 weeks old piglets, initial (T1) blood samples and rectal swabs were obtained from each the 60 piglets. These samples were analyzed in pools of two yielding a total of 30 pooled samples to determine the *Salmonella* seroprevalence and the proportion of pigs excreting *Salmonella*.

Blood samples were taken again when the pigs were 11 weeks old (T2) and at the end of the observation period, when the pigs were 23 weeks of age (T3). At the beginning of the observation period, each pig was weighed (W1). Weighing was repeated when the pigs were 9, 15, 17, and 23 weeks old (W2–W5) and these measurements were used to calculate the daily weight gain (DWG) using the formula: weight in kg gained/#days between weighing. Similar for feed conversion ratio (FCR), the following formula was used: kg consumed/weight in kg gained in the period. Feed consumption was estimated from the data sheet delivered to the farm manager and workers in charge of supplying feed to the pigs, and on which they noted the number of packages of feed supplied to each pen. The amount of feed in kg consumed by pigs in each pen and each group of pigs was then calculated.

### 2.4. Serological and microbiological analysis

The blood samples were stored and transported in a refrigerator (4–5°C) within 24 h after sample collection. Subsequently, serum was extracted and the ELISA diagnostic kit IDEXX^®^ Swine *Salmonella* Ab (IDEXX, Barcelona, Spain) was used to evaluate the seroprevalence of *Salmonella* spp., using a cut-off of 40% optical density.

The 30 pooled rectal swabs were duly marked and transported within 24 h at 4–5°C to the Veterinary and Zootechnical Laboratory of ICMT, which was in charge of processing and analyzing the samples. Upon arrival at the laboratory, the samples were inoculated into peptonized water at an adjusted ratio of 1:10 weight/volume and incubated at 36 ± 1°C for 18 ± 2 h after which 1 ml was incubated in selenite cystine broth and incubated at 36 ± 1°C for 24 ± 2 h. On day three, 0.1 ml of the broth was inoculated onto Xylose Lysine Deoxycholate (XDL; ICMT, Colombia) agar and Hecktoen agar (ICMT, Colombia) and incubated at 36 ± 1°C for 18 ± 2 h. Suspected *Salmonella* spp. colonies were selected from both agar media and re-streaked onto MacConkey agar (ICMT, Colombia) to obtain pure colonies after incubation at 36 ± 1°C for 18 ± 2 h. Subsequently, suspected isolates were tested by urea and sulfide-indole-motility tests as well as Gram staining. Finally, suspected isolates were subjected to PCR to confirm the serogroup and serotype, using the primers and conditions previously described by Cardona-Castro et al. (33).

### 2.5. Statistical analysis

Data from all pigs were used. The serological samples of the animals that died during the follow-up period were filled according to the mode of the results. For the statistical analyses, SPSS^®^ version 21 CES university license, Microsoft Office Excel 2003 (Microsoft Corporation, Redmond, USA), JAMOVI version 1.8.4 of free distribution and EPIDAT 3.1 of free distribution was used.

A univariate analysis was carried out to describe the distribution of pigs included in the study according to their sex, age, weight, *Salmonella* seroprevalence and the line (breeder or finisher). To check for normality of the distribution of quantitative variables, Shapiro-Wilk normality test was performed. Next, bivariate analyses were undertaken investigating the association between the different variables, with a focus on the effect of treatment. Parametric tests were used for dependent quantitative variables that were normally distributed (T-student test), whereas non-parametric tests were used for the non-normally distributed variables (Mann-Whitney U test). Chi-square test was used for the count data variables, and the Fisher exact test was used when one or more of the expected cell values were <5. For all analyses, the *P*-value was reported using a significance value of α = 0.05 (34, 35). Due to the limited number of samples, no attempts were made to model the seroprevalence over time using repeated measurements models.

## 3. Results

### 3.1. Salmonella baseline

In August 2020, the baseline seroprevalence of *Salmonella* was 59.0% in the pig herd. Only one *Salmonella-*positive sample was found and confirmed by PCR among the 100 fecal samples analyzed. The 130 fecal samples obtained between September 2020 and April 2021 were all negative for *Salmonella*. In 2021, the results of the second baseline sampling analyzing 100 blood samples yielded a seroprevalence of 47.0% (Table 1).

**Table 1:** *Salmonella* seroprevalence among the 100 pigs included in the base line study.

**Date**	**Type of pig**	**No. of animals**	**Age**	***Salmonella*** **serology positive samples (%)**	**Average seroprevalence**
10/8/20	Lactating sows	10	1 year	6 (60.0%)	59.0%
10/8/20	Weaned piglets	30	6 weeks	9 (30.0%)	
17/8/20	Growing pigs	30	13 weeks	21 (70.0%)	
17/8/20	Finishing pigs	30	22 weeks	23 (76.7%)	
26/10/21	Lactating sows	10	1 year	7 (70.0%)	47.0%
26/10/21	Weaned piglets	30	9 weeks	3 (10.0%)	
21/10/21	Growing pigs	30	13 weeks	18 (60.0%)	
21/10/21	Finishing pigs	30	22 weeks	19 (63.3%)	

### 3.2. Clinical trial

The distribution of the pigs according to sex, age, line, *Salmonella* prevalence and weight is presented in Table 2 and shows no statistical difference between the two groups. During the observation period, six animals died among including four pigs from the intervention group and two pigs in the control group (Supplementary Figure S1). Based on a necropsy examination, the pigs died due to infarction, hemorrhage, meningitis, intestinal torsion and pneumonia. Hence, the causes of death were not related to the water and feed additions and this level of mortality was normal at the farm.

**Table 2:** Descriptive analysis of characteristics and *Salmonella* seroprevalence of 120 weaned piglets included in the clinical trial with organic acids.

**Variable**	**Control group**		**Intervention group**		***P*** **-value for group difference**
	**frequency**	**Relative frequency**	**Absolute frequency**	**Relative frequency**	
**Sex**					
Female	35	58.3%	31	51.7%	0.22
Castrated	14	23.3%	10	16.7%	
Male	11	18.3%	19	31.7%	
**Line**					
Breeder	17	28.3%	12	20.0%	0.29
Finisher	43	71.7%	48	80.0%	
***Salmonella*** **seroprevalence**					
Positive at T1^a^	1	1.7%	1	1.7%	1
Positive at T2	28	47.7%	11	18.3%	<0.001
Positive at T3	46	76.7%	37	61.7%	0.075
**Variable**	**Median**	**IQR**	**Median**	**IQR**	***P*** **-value for group difference**
Age (days) at T1	42	3	42	2	0.97
Weight (kg) at T1	15	3	14	3	0.11
Total	60	100%	60	100%	

a T1–T3 is the three times that *Salmonella* seroprevalence was measured during the trial where T1 was at the beginning of the trial, when the pigs were 6 weeks old, T2 at 11 weeks of age, and T3 at 23 weeks of age.

At T1, when the pigs were 6 weeks of age a *Salmonella* seroprevalence of 1.7% was found in both groups. At T2, when the pigs were 11 weeks of age a *Salmonella* seroprevalence of 18.3% was observed in the intervention group vs. 47.7% in the control group, showing a statistically significant difference between groups (*P* < 0.001). Finally, at T3 where the pigs were 23 weeks of age a *Salmonella* non-significant seroprevalence of 61.7% was observed in the intervention group vs. 76.7% in the control group (*P* = 0.075) (Supplementary Figure S2).

**Figure 2:**
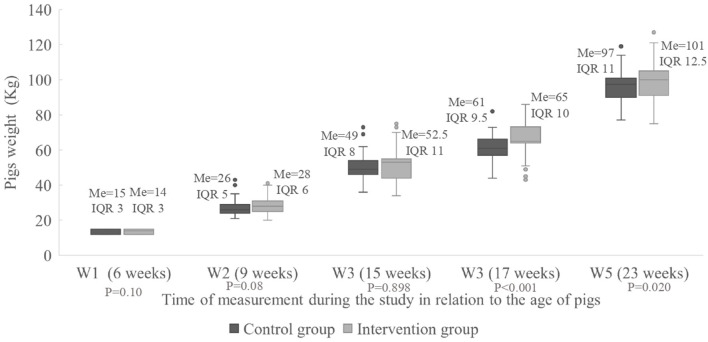
Weight of the individual pigs (kg) for the intervention and control groups measured five times during the study. Me, median; IQR, interquartile range; W1–5, Weight all time 1–5.

The median and the interquartile range (IQR) of the weight of the pigs at the different times of measurements are shown in Figure 2. There was a statistically significant difference between the groups at W4 (*P* < 0.001) where the pigs were 17 weeks old with a better performance in the intervention group, where the median weight was 65.0 kg per pig (IQR = 10.0 kg) vs. 61.0 kg in the control group (IQR = 9.5 kg). Likewise, at W5 where the pigs were 23 weeks old, the growth performance was significantly higher (*P* = 0.024) in the intervention group, where the median weight was 101.0 kg per pig (IQR 12.5 kg) vs. 97.0 kg in the control group (IQR 11.0 kg).

**Figure 3:**
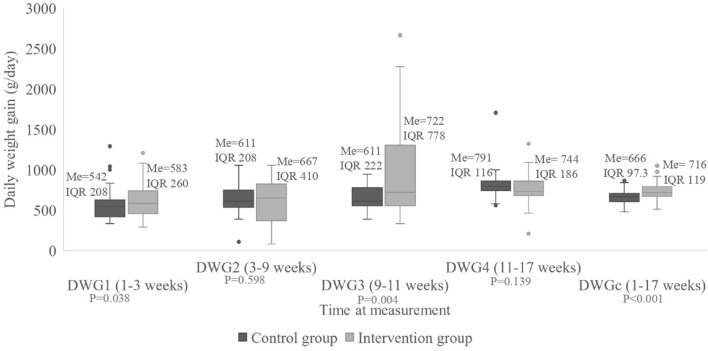
Daily weight gain of the pigs (g/day) divided according to group, measured at four times during the study as well as for the entire period. Me, median; IQR, interquartile range; DWGc, cumulative daily weight gain.

For DWG3, there was a statistically significant difference between treatment groups (*P* < 0.001), showing higher values in the intervention group, which had a median of 722 g/pig/day (IQR 22 g/pig/day) vs. a median of 611 g/day (IQR 78 g/pig/day) in the control group (Figure 3). There was no difference between groups for DWG1, DWG2, and DWG4. However, the median of the cumulative DWG was 743 g/pig/day (IQR 12 g/pig/day) for the intervention group vs. 666 g/pig/day (IQR 10 g/pig/day) for the control group, showing a statistically significant difference (*P* < 0.001).

**Figure 4:**
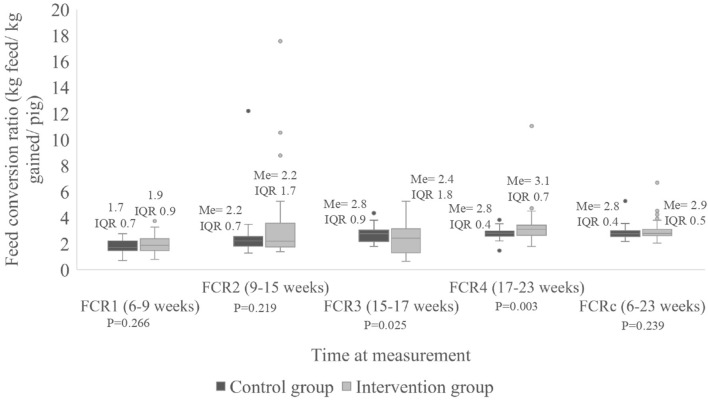
Feed conversion ratio (FCR) per pig (in kg/kg gained/pig) divided according to group, measured at four times during the study as well as for the entire period. Me, median; IQR, interquartile range; FCRc, cumulative feed conversion ratio.

Regarding FCR, a statistically significant difference (*P* = 0.025) was observed at FCR3 where a median of 2.4 kg of feed per kg of weight gained (IQR 1.8 kg) was estimated for the intervention group vs. 2.8 kg (IQR 0.9 kg) in the control group. For FCR4, a statistically significant difference (P = 0.009) was observed where a median of 3.1 kg of feed per kg of weight gained (IQR 0.7 kg) was estimated for the intervention group vs. 2.8 kg (IQR 0.4 kg) in the control group (Figure 4). However, there was no significant difference (P = 0.14) when the median cumulative FCR was compared between groups, as the pigs in the intervention group used 2.8 kg of feed per kg weight gained (IQR 0.6 kg) vs. 2.7 kg of feed (IQR 0.4 kg) the control group.

In the intervention group, the total feed consumption was 13,120 kg vs. 12,680 kg in the control group (*P* = 0.61). This corresponded to an average feed consumption of 2 kg per animal per day for the intervention group and 1.9 kg for the control group (*P* = 0.87). Furthermore, the total water consumption for the intervention group was 78,144 L and for the control group 70,310 L. The water consumption variable was not normally distributed; the median consumption was 620 L/day (IQR 460) for the intervention group and 560 L of water/day (IQR 410) for the control group. The difference in water consumption was not statistically significant (*P* = 0.09).

## 4. Discussion

The baseline results showed a high *Salmonella* seroprevalence of 59.0%, which did not concord with the low proportion of pigs excreting the bacteria as shown by the culture-based detection method (1.0%) (36). To investigate this further, several methods and growth media were used to increase the sensitivity (37). However, these efforts were not successful in increasing the number of *Salmonella*-positive samples. This may be because of the known low sensitivity of culturing *Salmonella* spp. in fecal samples from pigs (8, 32, 38–40). Moreover, the regular administration of growth promoters to piglets on the farm could have reduced the *Salmonella* spp. excretion (40). Therefore, it was decided to measure only *Salmonella* seroprevalence during the study as an indication of the *Salmonella* prevalence.

There was a significantly lower *Salmonella* seroprevalence in the group of pigs provided organic acids (OA) (18.3%) compared with the control group (47.7%) at T2 (11 weeks). Contrary, at T1 (6 weeks) and T3 (15 weeks), there was no statistical difference in seroprevalence. OA favor the growth of lactobacilli, which contributes to a low pH, limit bacterial growth in the intestines and stimulates the immune system in a non-specific way; all of which decrease the probability of *Salmonella* colonization (3, 22, 41, 42). For this reason, the use of OA may have delayed the excretion and spread of *Salmonella* during the post-weaning period. However, as the observation period progressed, the majority of the pigs were eventually exposed to *Salmonella* spp. at some point. These findings are in agreement with the literature (3, 41, 43). Pigs develop partial immunity to *Salmonella* when the spread and exposure to the pathogen is reduced. Such partial immunity development is the core of a *Salmonella* reduction strategy as pigs at the time of slaughter will have a lower probability excreting *Salmonella* (36, 44). It is well-known that *Salmonella* cannot be eradicated without culling the farm (36, 44). There is a positive association between herd serology and the prevalence of *Salmonella* on the carcass as a low seroprevalence is associated with less prevalence on the carcass, less excretion and less overall contamination with *Salmonella* at the abattoir (45).

In Colombia, it is customary practice to use growth promoters like tylosin and zinc bacitracin. However, this is not in line with the principles of prudent use as it will lead to development of AMR and growth promoters are now banned in many countries (46, 47). Growth promoters are used as they are believed to support increased growth and reduce the severity of post-weaning diarrhea. In our study, pigs provided OA had a better cumulative DWG and weight productively than pigs administered growth promoters. The OA are feed additives that are metabolized by the animal, allowing their use without the risk of residues accumulating in the meat. The use of OA is already increasing as a response to strengthened regulations and consumer concerns on the use of antimicrobials in many countries (48). The mode of action of OA includes modulating stimulus that benefits the development of the mucosa, the length of microvilli, intestinal cell growth and therefore the absorptive capacity of the intestine is improved (3, 19).

The weight measured at time points W4 (17 weeks) and W5 (23 weeks) and the cumulative DWG during the study showed a better growth performance of pigs administered OA compared with the control group, which supports findings in other studies (49–56). van der Heijden et al. concluded that Selko pH^®^ added to water at a concentration of 0.2% significantly reduced the seroprevalence of *Salmonella* and improved the productive performance of pigs (57). Likewise, formic, citric and benzoic acids can lead to improved growth when added to feed provided to weaned and growing pigs (23). A better DWG translates into less time spent by the pig in the herd, as well as a more efficient use of feed nutrients that represent one of the main costs of production (58).

Although there was a statistically significant difference (*P* = 0.025) at FCR3 and FCR4 (*P* = 0.009), favoring the intervention group and control group respectively, there was no significant difference in the cumulative FCR between the two groups. This may because the staff in charge of supplying the feed to the pigs did not fully take into account the pigs that died during the trial when calculating the feed to be administered. Therefore, the number of pigs used to calculate the feed provided was likely a little too high which may explain that no difference was found in the cumulative FCR between the groups (59).

The cleaning of the water pipes on the farm with citric acid before and during the study improved the water quality, which likely also resulted in healthier pigs (60, 61). Water is a potential source of various pig pathogens causing diseases that affect weight gain and feed conversion (62). For this reason, it is recommended to clean the water pipes regularly. The combination of cleaning of the pipes and the use of OA may be responsible for the higher overall productivity and apparently slower spread of *Salmonella* in the group administered OA. At the abattoir, such pigs are expected to have a lower probability of excreting *Salmonella* (44).

*Salmonella* antibodies can remain at measurable levels up to 3 months in the pig, which means that positives animals can be found even when they no longer are infected or excreting *Salmonella* spp. (36). Pigs included in the clinical trial may have experienced exposure to the pathogen without excreting *Salmonella* during sampling. Additionally, presence of antibodies in the individual animal may not be directly related to a carrier stage or probability of shedding *Salmonella* spp. (63). Hence, it is a limitation of our study that no other diagnostic tests were applied that could confirm whether pigs were excreting *Salmonella* (13). Post-harvest sampling of lymph nodes and ileocecal contents of the pigs may have increased the likelihood of detecting *Salmonella* if present, and thereby allowing a better assessment of how OA impacted the *Salmonella* levels in the pigs (37).

Selacid^®^ was supplied at different concentrations during the study. It is known that different concentrations of OA can affect the *Salmonella* seroprevalence in pigs as shown by Calveyra et al. who concluded that at a concentration of 0.1%, OA had no significant effect on the *Salmonella* level whereas it did have a significant effect on improving daily weight gain in the pigs (64).

The dosage of Selko pH^®^ we administered to drinking water (0.8 ml/L) was slightly lower than the dosage recommended by the technical data sheet (1–2 ml/L) from the manufacturer (65). The total estimated cost of the growth promoters added to the administered feed was 32 US$ as compared to 57 US$ for OA added to water and feed. The relative low dosage of OA may have had a reduced effect on *Salmonella* in the intervention group (25, 30, 66). On the other hand, the additional supplement of OA in the feed probably compensated for the lower concentration of Selko pH^®^ used in the water. The types and concentrations of different OA products—as well as their costs—should be further investigated for their effect on *Salmonella* and overall productivity as the effect of the acids varies significantly depending on the components present in the feed (23, 59, 63). Moreover, attention should be given to palatability of the OA to ensure that the pigs do not consume less water or feed. Contrary to traditional organic acids, Selko pH^®^ has the advantage that it is safe to use as it can be given to pigs in relative high concentrations without risking that the pigs stop drinking because of palatability issues.

For future research, it is recommended to include pig farms with known high prevalence of *Salmonella* spp., serial sampling and analyses of 25-g of fecal samples to increase the sensitivity.

## 5. Conclusion

This pilot study indicates that administration of OA in combination with regular cleaning of water pipes can improve productivity and delay exposure to *Salmonella* spp. when compared with commonly used antimicrobial growth promoters. A substitution of antimicrobial growth promoters with OA will lower antimicrobial use and resistance, while ensuring productivity. However, the study should be repeated before firmer conclusions can be drawn regarding productivity and the *Salmonella* spp. reduction potential of OA.

## Data availability statement

The raw data supporting the conclusions of this article will be made available by the authors, without undue reservation.

## Ethics statement

This study was endorsed by the Institutional Committee Cuidado y Uso de los Animales (CICUA) at the CES University, Medellin, Colombia (Code No. 206/Act No. 38).

## Author contributions

AD, LA, and MR-H were responsible for the conception of the study, experimental design, and manuscript writing. MR-H was responsible of collection of samples in the farm, data analysis, and interpretation. NC-C, LR-R, and LV-A were responsible for conception of the study and experimental design. All authors contributed to the article and approved the submitted version.
