# Supplementary material for: Pilot study of the productivity and Salmonella seroprevalence in pigs administered organic acids
Source: Front Vet Sci. 2023 Mar 3;10:1123137. doi: 10.3389/fvets.2023.1123137 (PMC10020582; doi:10.3389/fvets.2023.1123137)
Supplement: Supplementary file 1 [file Table_1.DOCX]

Supplementary Material

Productivity and *Salmonella* seroprevalence in pigs administered organic acids

Manuela Roldan-Henao^1*^, Anders Dalsgaard^2^, Nora Cardona-Castro^3^, Lina Restrepo-Rivera^3^, Luis Veloza-Angulo^1^ Lis Alban^2,4^

^1^ Department of Veterinary and Animal Sciences, CES University, Medellin, Colombia

^2^ Department of Veterinary and Animal Sciences, University of Copenhagen, Copenhagen, Denmark

^3^Instituto Colombiano de Medicina Tropical, CES University, Medellin, Colombia

^4^Department for Food Safety and Veterinary Issues, Danish Agriculture & Food Council, Copenhagen, Denmark

*** Correspondence:**

Manuela Roldan-Henao
maroldanh@uces.edu.co

# Supplementary Figures and Tables

Table S1. Composition of the feed given to the pigs during the study

| Type of pig | Component | Concentration |
| --- | --- | --- |
| Weaner^a^ | Protein | 20% |
|  | Fiber | 4% |
|  | Energy | 3300 Kcal |
| Growing^b^ | Protein | 17% |
|  | Fiber | 5% |
|  | Energy | 3200 Kcal |
| Finisher^c^ | Protein | 15% |
|  | Fiber | 6% |
|  | Energy | 3200 Kcal |

^a^ Feed ingredient: maize, extruded soybeans, fish fluor and choline chloride

^b^ Feed ingredients: maize, soybean fluor, extruded soybeans and choline chloride

^c^ Feed ingredients: maize, soybean fluor and choline chloride
