# Supplementary figures and images for: Pilot study of the productivity and Salmonella seroprevalence in pigs administered organic acids
Source: Front Vet Sci. 2023 Mar 3;10:1123137. doi: 10.3389/fvets.2023.1123137 (PMC10020582; doi:10.3389/fvets.2023.1123137)

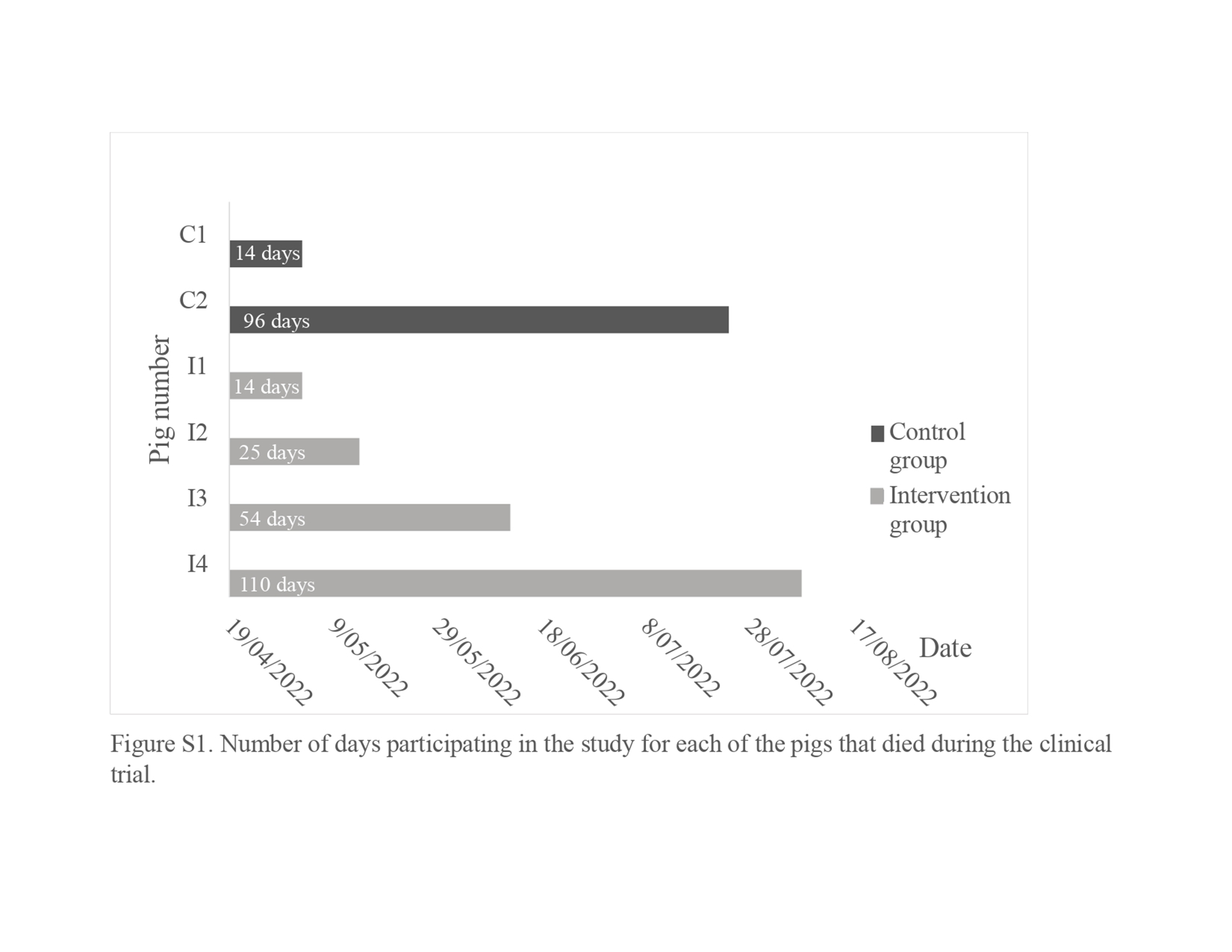

Supplement: Supplementary file 2 [file Image_1.JPEG]

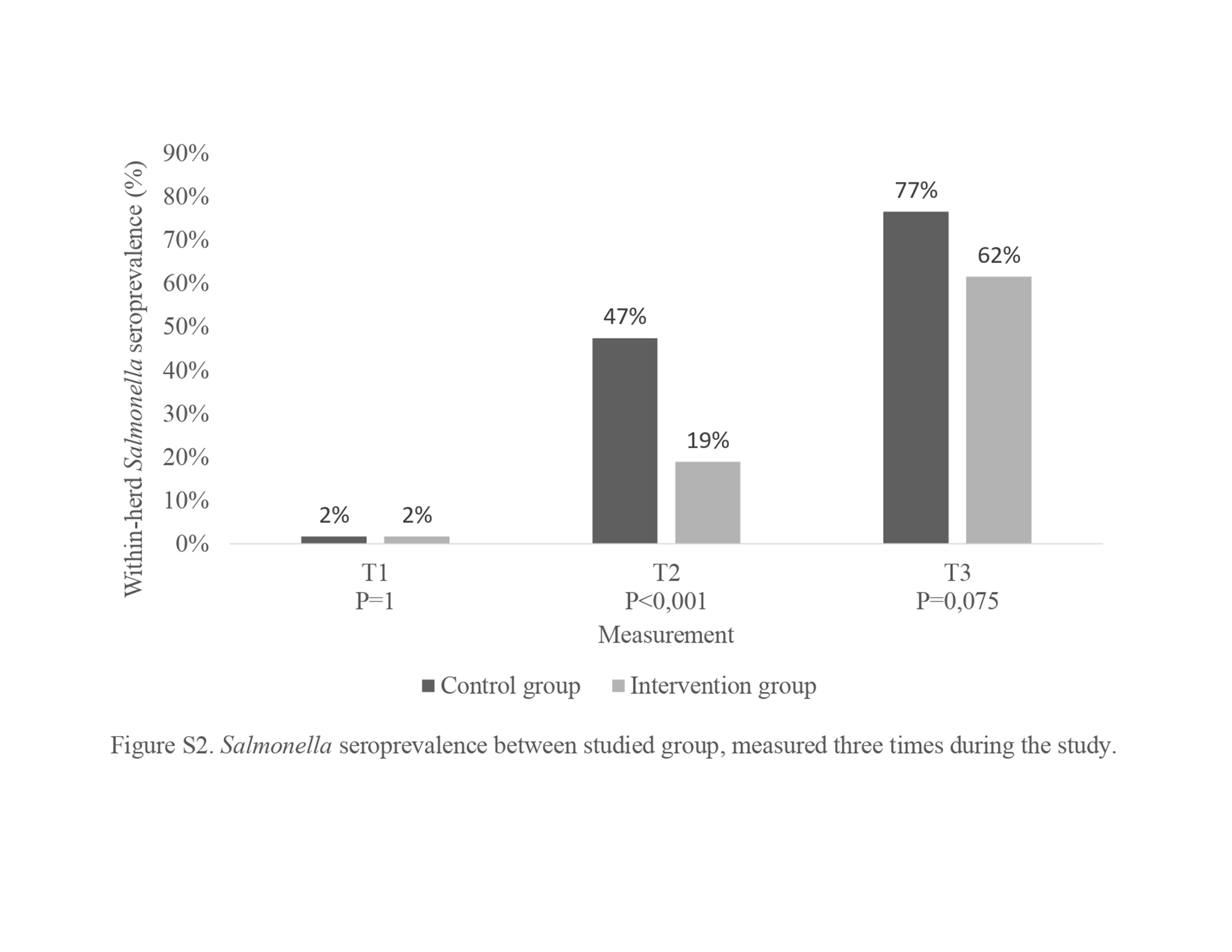

Supplement: Supplementary file 3 [file Image_2.JPEG]
